# Spheroid Culture of Head and Neck Cancer Cells Reveals an Important Role of EGFR Signalling in Anchorage Independent Survival

**DOI:** 10.1371/journal.pone.0163149

**Published:** 2016-09-19

**Authors:** Diana Braunholz, Mohammad Saki, Franziska Niehr, Merve Öztürk, Berta Borràs Puértolas, Robert Konschak, Volker Budach, Ingeborg Tinhofer

**Affiliations:** 1 Translational Radiooncology and Radiobiology Research Laboratory, Department of Radiooncology and Radiotherapy, Charité University Hospital Berlin, Berlin, Germany; 2 German Cancer Consortium (DKTK), Deutsches Krebsforschungszentrum (DKFZ), Partner Site Berlin, Charité University Hospital Berlin, Berlin, Germany; Chang Gung University, TAIWAN

## Abstract

In solid tumours millions of cells are shed into the blood circulation each day. Only a subset of these circulating tumour cells (CTCs) survive, many of them presumable because of their potential to form multi-cellular clusters also named spheroids. Tumour cells within these spheroids are protected from anoikis, which allows them to metastasize to distant organs or re-seed at the primary site. We used spheroid cultures of head and neck squamous cell carcinoma (HNSCC) cell lines as a model for such CTC clusters for determining the role of the epidermal growth factor receptor (EGFR) in cluster formation ability and cell survival after detachment from the extra-cellular matrix. The HNSCC cell lines FaDu, SCC-9 and UT-SCC-9 (UT-SCC-9P) as well as its cetuximab (CTX)-resistant sub-clone (UT-SCC-9R) were forced to grow in an anchorage-independent manner by coating culture dishes with the anti-adhesive polymer poly-2-hydroxyethylmethacrylate (poly-HEMA). The extent of apoptosis, clonogenic survival and EGFR signalling under such culture conditions was evaluated. The potential of spheroid formation in suspension culture was found to be positively correlated with the proliferation rate of HNSCC cell lines as well as their basal EGFR expression levels. CTX and gefitinib blocked, whereas the addition of EGFR ligands promoted anchorage-independent cell survival and spheroid formation. Increased spheroid formation and growth were associated with persistent activation of EGFR and its downstream signalling component (MAPK/ERK). Importantly, HNSCC cells derived from spheroid cultures retained their clonogenic potential in the absence of cell-matrix contact. Addition of CTX under these conditions strongly inhibited colony formation in CTX-sensitive cell lines but not their resistant subclones. Altogether, EGFR activation was identified as crucial factor for anchorage-independent survival of HNSCC cells. Targeting EGFR in CTC cluster formation might represent an attractive anti-metastatic treatment approach in HNSCC.

## Introduction

Each day millions of tumour cells are shed into the blood circulation from solid tumours [1]. Of these cells, only a small subpopulation is able to survive and demonstrates tumour-inducing potential enabling metastastic progression [2,3]. Circulating tumour cells (CTCs) have been detected in peripheral blood of patients in most epithelial tumour types and were significantly associated with poor prognosis [4–9]. Previous findings revealed the existence of so-called CTC clusters or circulating microembolis (CTM) which display an increased metastatic potential compared to solitary CTCs [10,11]. In agreement with this, *in vivo* spheroids were shown to be exclusively detectable in blood from patients with metastatic disease in various histological entities indicative of their role in tumour progression and metastasis [12]. CTC clusters can be built from CTCs alone or are mixed with accessory cells including leukocytes, platelets, endothelial cells or fibroblasts [13–15]. In contrast to solitary CTCs, these CTC aggregates (e.g. ≥ 3 CTCs in advanced NSLCLC) [16] were shown to have an advantage in the blood circulation in terms of protection from an immune attack and anoikis (apoptosis resulting from loss of cell–cell and cell–matrix contact) [14,17]. Identification of the molecular mechanisms underlying the CTC cluster formation ability and their maintenance in the blood circulation may lead to a better understanding of the mechanisms involved in the metastatic potential of CTCs and might identify novel therapeutic targets for anti-metastatic treatment.

In the seminal study of Jost and coworkers, EGFR activation was identified as key factor for anchorage-independent cell survival of primary and immortalized human keratinocytes [18]. Subsequent studies demonstrated this function of EGFR in different epithelial tumour models as well [19–21]. EGFR is overexpressed in many tumours of epithelial origin including HNSCC showing upregulated expression in about 90% of patients [22]. Increased levels of EGFR expression and activation have been associated with poor prognosis, distant metastasis, and therapy resistance [23]. We have previously shown in a breast xenograft model that EGFR as well as mesenchymal markers are upregulated in the CTC fraction [24]. Additionally, in HNSCC patients with locally advanced disease, we have detected EGFR in the total fraction of CTCs and its phosphorylated form in more than 50% of CTCs [25]. However, the causative role of EGFR and its downstream signalling pathway for anchorage-independent cell survival of CTCs in HNSCC remains unresolved. Previous studies established the forced suspension culture as a near-physiological *in-vitro* model in which the tumour spheroids rather than cells cultured in monolayers mimic biological properties of micrometastases/CTC clusters, including their architecture as well as morphological and physiological characteristics [26]. In the current study, we used the spheroid model mimicking the conditions of these CTC clusters and anchorage-independent growth to evaluate whether signalling from EGFR may affect their survival and cluster formation ability in the absence of matrix engagement.

## Materials and Methods

### Reagents

Cetuximab (CTX) was provided by Merck Serono (Darmstadt, Germany) and unless otherwise indicated CTX was used at a concentration of 100μg/ml. Recombinant epidermal growth factor (EGF) and amphiregulin (AREG) were purchased from Invitrogen (Carlsbad, CA, USA). These reagents were used at a concentration of 100ng/ml (EGF, AREG). Gefitinib was purchased from SelleckChem (Houston, Tx, USA) and used at a concentration of 100nm.

### Cell Lines

The previously established HNSCC cell lines UT (University of Turku)-SCC-9 (UT-SCC-9) and SCC-9 cells were kindly provided by T.K. Hoffmann (University of Essen, Dept. of Otorhinolaryngology). The FaDu cell line was purchased from ATCC (Cat. No: ATCC® HTB-43™). The identity of the cell lines was confirmed by high-throughput SNP-based authentication (Multiplexion, Heidelberg, Germany). CTX-resistant subclone of UT-SCC-9 (UT-SCC-9R) was established by chronic treatment of UT-SCC-9P cells with increasing concentrations of CTX from 50μg/ml up to 200μg/ml over a period of 7 months. The resistance of treated cells was validated by performing clonogenic assays (S1 Fig). In addition, UT-SCC-9R was verified to originate from the UT-SCC-9P by next-generation sequencing in our lab. Cells were cultured in MEM supplemented with 10% fetal calf serum (FCS) and incubated in a humidified atmosphere of 95% air / 5% CO_2_ at 37°C. All cell lines were routinely tested for mycoplasma contamination.

### Spheroid Cell Culture

Non-adhesive tissue culture plates were used for forced suspension culture. Briefly, the anti-adhesive polymer poly-HEMA (Sigma Chemical Co., St. Louis, MO, USA) was dissolved in ethanol to a final concentration of 10mg/ml and used for coating tissue culture plates. The ethanol was evaporated overnight at room temperature and plates were sterilized under UV light for 2h. Thereafter, cells were plated into pre-coated dishes in serum-free medium unless stated otherwise. For assessment of the spheroid formation capacity, HNSCC cells were seeded at a density of 2,500 cells/well in non-adhesive 96-well plates. After 96h, the total number of spheroids per well was determined under light microscope.

For analysis of the effects of EGFR stimulation or blockade on spheroid volume, HNSCC cells were seeded at a density of 300,000 cells in non-adhesive 6-well plates. Cells were left untreated or were treated with AREG, EGF, CTX or gefitinib. After an incubation of 72h at least two microscopic images were taken of each culture well from the areas with highest spheroid density. To calculate the mean spheroid volume short and long diameters of 15 spheroids per sample were measured by the software Image J [27]. Spheroid volumes were then calculated according to the formula V = *a*×*b*^2^×π/6, with *a* and *b* representing the lesser and greater spheroid diameters, respectively. Relative spheroid volumes were calculated by setting volumes of non-treated spheroids to 100%.

### Clonogenic Survival Assay

To analyse clonogenic survival following the culture of HNSCC cells in forced suspension, spheroids were disaggregated to single cells by 10-min treatment with trypsin (0.25%)/EDTA. Thereafter, cells were plated into 12-well plates at a density of 250 cells per well and incubated until colonies were formed. Colonies containing 50 cells or more were counted. Plating efficiency and survival fractions for given treatments were calculated on the basis of survival of non-treated cells. All samples were done in triplicates and at least three independent experiments were carried out.

### Proliferation Assay

The growth kinetics of HNSCC cells was determined by cell counting. To this aim, 2×10^4^cells/well were plated into 12-well culture plates in MEM containing 10% FCS. On days 1, 2, 3 and 4, cells were harvested by trypsinisation and counted in the haemocytometer under the light microscope.

### Apoptosis Assay

For the detection of apoptotic cells, samples from the volume measurements were harvested by trypsinisation and analysis of apoptosis was performed by propidium iodide (PI) staining followed by flow cytometric analysis as described by Riccardi and Nicoletti [28]. Briefly, cells were fixed by 70% (v/v) cold ethanol and stored overnight at -20°C. Fixed cells were washed with PBS and stained using DNA staining solution (20μg of PI in 1 ml of PBS containing 2mg of RNase). Subsequently, the fraction of cells with a DNA content less than 2n ("sub-G_1_ cells") as a result of apoptotic DNA fragmentation was determined using a FACSCanto II cytometer (BD Biosciences Europe, Heidelberg, Germany). For each sample 10,000 events were recorded. Data analysis was performed with BD FACSDiva Software v6 (BD Biosciences).

### MTT Cell Viability Assay

Cells were seeded in coated or non-coated 96-well plates at the optimal cell concentration for each cell line (10,000–75,000 cells (forced suspension); 2,500 cells (monolayer)). Cells were then left untreated or were treated with AREG, EGF, CTX or gefitinib. Seventy-two hours after seeding, the MTT reagent (5mg/ml) was added and cells were incubated overnight. After addition of DMSO, absorbance at 540nm was measured by the use of the AR 2001 microplate reader (Anthos Mikrosysteme GmbH, Krefeld, Germany). The relative cell viability was calculated using untreated cells as control.

### Western Blotting

Expression levels of phosphorylated-MAPK/ERK (42/44 kDa) were assessed by Western blotting. Cells were treated as indicated in the figure legends. After treatment, cells were lysed with RIPA buffer (Thermo Scientific, Schwerte, Germany) and protein concentrations were measured. Thereafter, proteins were electrophoretically separated by SDS-PAGE and transferred to PVDF membranes (BioRad, Munich, Germany). Membranes were incubated with the specific antibodies for pERK1/2 followed by incubation with a secondary antibody conjugated to horseradish peroxidase (Cell Signalling Technology, Danvers, MA, USA). Immuno reactive proteins were visualized using enhanced chemiluminescence (WesternSure® PREMIUM Chemiluminescent Substrate, LI-COR, Bad Homburg, Germany) and the C-Digit Blot scanner (LI-COR, Bad Homburg, Germany). For loading control an antibody against vinculin (124 kDa; Abcam, Cambridge, UK) was used.

### Statistical Analysis

For assessment of the effects of EGFR stimulation and EGFR blockade on spheroid formation capacity, apoptosis, proliferation and clonogenic survival, the paired Student`s *t*-test was used. The level of significance was set at *p* < 0,05. Statistical analyses were carried out using the SPSS Statistics software (version 22.0.0, IBM, Armonk, NY, USA).

## Results

### The Extent of Spheroid Formation is Cell Line-Dependent

**Fig 1:**
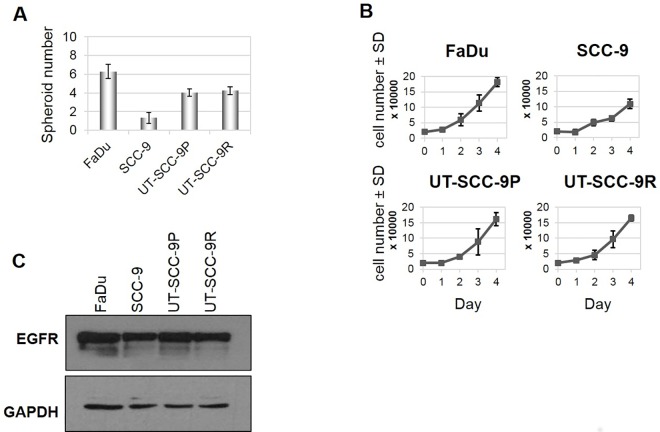
Spheroid generation is cell line dependent. (A) 2,500 cells were seeded into 96-well plates (poly-HEMA coated). After 96h, total number of spheroids per well were counted. (B) 2 ×10^4^cells/well were plated into 12-well culture plates and at indicated times cells were counted. The data points shown in (A) and (B) represent the mean values ± standard deviation (SD) of three independent experiments. (C) Cells were lysed, and protein samples were subjected to western blot analysis with specific antibodies against EGFR. GAPDH was used as a loading control.

Since established HNSCC cell lines can show different properties in cell growth and invasiveness, we first assessed the extent of spheroid formation in forced suspension in relation to the growth rates of HNSCC cell lines in monolayer cultures. In the four HNSCC cell lines tested, we observed formation of spheroids from single-dissociated HNSCC cells in forced suspension cultures. As depicted in Fig 1A, FaDu cells showed the highest and SCC-9 cells the lowest potential to form spheroids, whereas no significant difference was observed in UT-SCC-9P cells compared to their cetuximab-resistant subclone UT-SCC-9R. (Fig 1A) The capability of HNSCC cells to form spheroids was positively correlated with their growth rates (Fig 1B) and the basal EGFR expression determined by immunoblotting. (Fig 1C)

### Autocrine EGFR Signalling Is Maintained in Spheroid Culture of HNSCC Cell Lines

**Fig 2:**
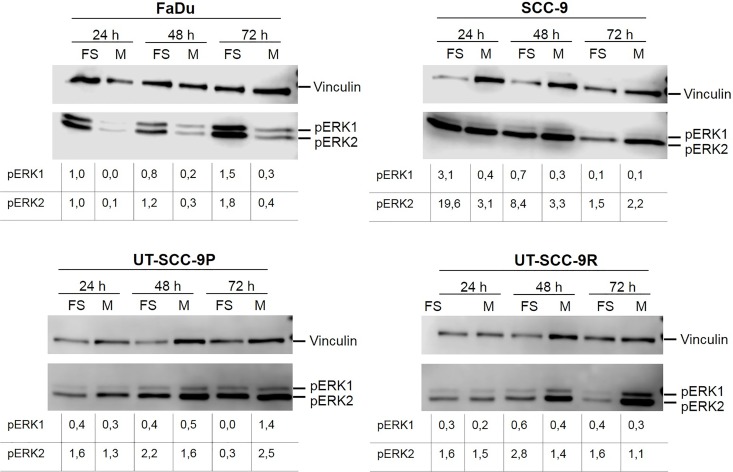
Autocrine EGFR signalling is maintained in spheroid culture of HNSCC cell lines. Cells were cultured as monolayer (M) or spheroids (FS) for 24h, 48h or 72h in serum-free medium. The expression levels of pERK1/2 (42/44 kDa) and vinculin (internal loading control; 124 kDa) were analysed by immunoblotting. The values of the quantitative analysis are depicted as pERK1/2 protein levels relative to vinculin expression levels.

Since it was shown that EGFR activity has protective effects on epithelial cells when growing in absence of matrix interaction, we investigated whether endogenous EGFR signalling was maintained under forced suspension conditions. As readout we used the activation of ERK1/ERK2 as downstream factor of the EGFR signal cascade, by examining its phosphorylation status (pERK1/2). All four cell lines were cultured either in monolayers or forced suspension. We used serum-free culture conditions for these experiments to avoid stimulation of EGFR by potential traces of exogenous EGFR ligands in foetal serum. Cells were harvested after 24h, 48h and 72h, and immunoblotting was performed. Activation of EGFR signalling was observed under both culture conditions in all cell lines (Fig 2). However, both the pattern of ERK1/ERK2 activation and its kinetics differed between the cell lines. Whereas FaDu demonstrated higher expression levels of pERK1 and pERK2 in forced suspension compared to monolayer cultures, the other three cell lines showed mainly pERK2 expression irrespectively of the type of culture condition. Interestingly, cell lines UT-SCC-9R and SCC-9 showed a marked reduction in pERK1/2 after 72h of spheroid culture. This can be explained by a declined growth and size of spheroids, which is not observed in the other two cell lines.

### Spheroid Formation Is EGFR-Dependent

**Fig 3:**
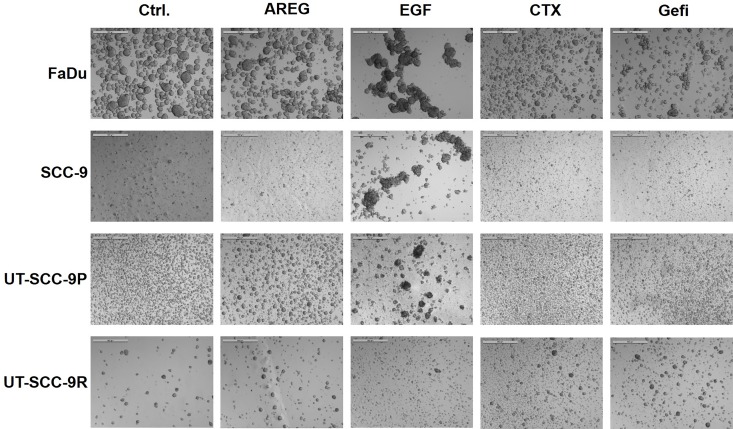
Effects of EGFR activation/blockade on spheroid formation. Cells were seeded at a density of 300,000 cells/well into 6-well plates and cultured under non-adherent conditions in the absence or presence of EGFR ligands (AREG, EGF) or EGFR blocking agents (CTX, gefitinib). Pictures were taken 72h after cell seeding. (5x magnification)

**Fig 4:**
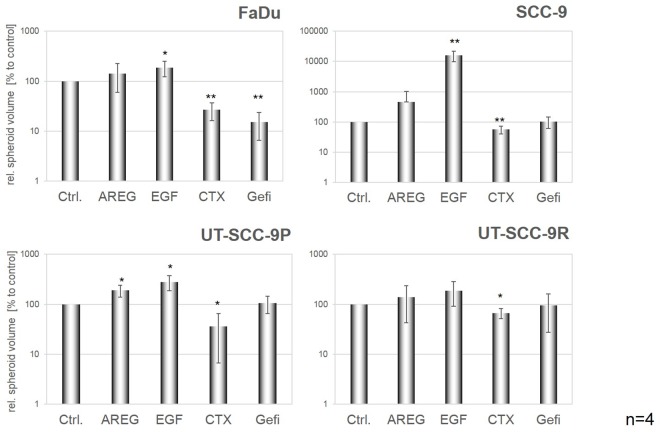
Effects of EGFR activation/blockade on spheroid volume. Cells were seeded at a density of 300,000 cells/well into poly-HEMA coated 6-well plates and treated with the indicated agents. After 72h, the diameters of at least 15 spheroids were measured and spheroid volumes calculated relative to the untreated spheroids. Bars represent mean values ± SD of four independent experiments. For the cell line SCC-9 the y-axis was adapted due to the EGF-induced strong increase in spheroid volumes.

To further explore the influence of EGFR signalling on the potential of HNSCC cells to escape anoikis and to survive in forced suspension, the four cell lines were either treated with EGFR ligands (100ng/ml AREG or EGF), the EGFR-blocking antibody cetuximab (CTX, 100μg/ml) or the EGFR-specific tyrosine-kinase inhibitor gefitinib, for 72h. Subsequently, the volume of formed spheroids was determined. In Fig 3, representative microscopic images from one of four independent experiments and from each cell line after activation or blockade of EGFR are shown. Especially in the cetuximab sensitive cell lines, an effect of either EGFR stimulation or EGFR blockade was observed. In Fig 4, the results from the quantitative analysis of volume measurements from four independent experiments are presented. For a better comparison of treatment effects, relative spheroid volumes were calculated in each experiment. Stimulation of cells cultured under non-adhesive conditions especially with EGF significantly increased spheroid volumes in the three CTX-sensitive HNSCC cell lines compared to their controls. Conversely, blocking of EGFR by CTX caused a markedly reduction of spheroid volumes in all cell lines. The effects of EGFR activation/blockade was more pronounced in FaDu and SCC-9 compared to UT-SCC-9P and UT-SCC-9R cells. Comparable effects were observed using gefitinib for EGFR blockade (Fig 4).

### EGFR Activation Has Protective Effects against Anoikis

**Fig 5:**
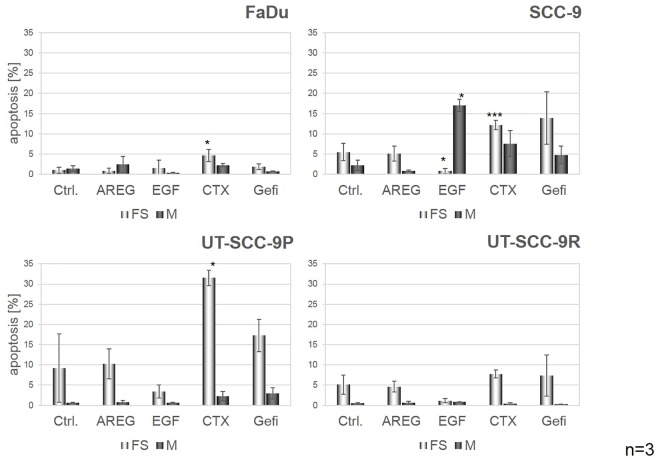
Activation of EGFR protects HNSCC cells against anoikis. Cells were seeded at a density of 300,000 cells/well in non-coated or poly-HEMA coated 6-well plates and were immediately treated with either EGFR ligands or blocking reagents. After 72h, cells from monolayer (M) and forced suspension (FS) cultures were harvested and the apoptotic fraction was determined by PI staining and subsequent flow cytometry. Bars represent the mean percentages of apoptotic cells ± SD of at least three independent experiments. Significant changes compared to control are marked with an asterisk.

**Fig 6:**
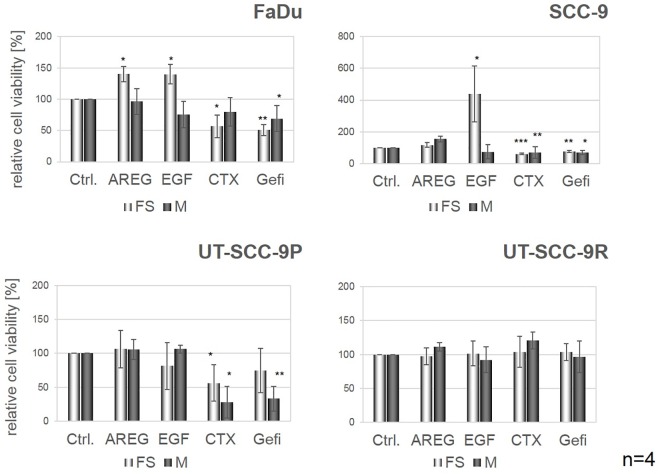
CTX affects proliferation of spheroids in sensitive cell lines. MTT assays were performed in 96-well plates with cells treated with EGFR stimulating or blocking reagents in monolayer (M) or forced suspension (FS) culture. Bars represent mean percentages ± SD of four independent experiments. Significant changes compared to control are marked with an asterisk.

We next evaluated whether cell survival of HNSCC cells in spheroids was also controlled by EGFR. For this purpose, all four cell lines were seeded and treated with AREG, EGF, CTX or gefitinib for 72h in forced suspension or monolayer cultures. In all three CTX-sensitive cell lines, reduced apoptosis was observed in EGFR ligand-treated compared to untreated cells (Fig 5). Although EGFR activation/blockade showed similar effects in forced suspension and monolayer cultures, the most prominent reduction or increase in apoptosis was observed in spheroid cultures, indicative for a stronger dependence of cells on EGFR signalling under the former culture conditions. Especially the cell line SCC-9 with the lowest capability to form spheroids demonstrated the strongest rescue in forced suspension culture after stimulation by EGF. Surprisingly, the monolayer showed reverse effects in this cell line. No significant changes were observed in UT-SCC-9R cells (Fig 5). These results were verified using MTT assays. (Fig 6) Corroborating the results from the analysis of apoptosis, a strong protective effect of EGFR stimulation in FaDu and SCC-9 cells cultured in forced suspension was observed. In UT-SCC-9P cells only slight effects of EGFR stimulation but clear effects of EGFR blockade were detected. In UT-SCC-9R cells EGFR stimulation or inhibition did not affect cell proliferation (Fig 6).

### Clonogenic Potential of HNSCC Spheroids Is Conserved by EGFR Activation during Forced Suspension Culture

**Fig 7:**
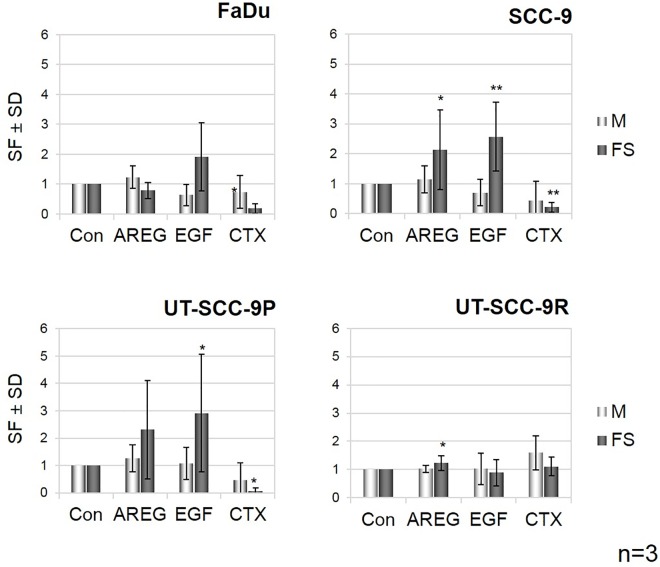
EGFR signalling regulates clonogenic survival of HNSCC cells derived from forced suspension cultures. Cells were cultured as monolayer (M) or in forced suspension (FS) in the absence or presence of EGF, AREG or CTX. 72h later, cells were harvested, disaggregated to single cells and subjected to clonogenic survival analysis. Bars represent the surviving fractions (SF) ± SD from three independent experiments. Significant changes compared to control are marked with an asterisk.

During their journey through hematogenous routes, small CTC clusters need to maintain their clonogenic potential for establishment of new lesions at distant organs. We therefore determined whether EGFR stimulation during forced suspension culture would also conserve the clonogenic potential of spheroid-derived HNSCC cells. Clonogenic survival assays were performed using cells which had been cultured for 72h either in monolayers or forced suspension in the absence or presence of EGFR ligands or CTX. Overall, clonogenic survival potential was slightly higher in cells derived from monolayer compared to forced suspension cultures. (Fig 7) Addition of AREG and EGF during forced suspension culture improved clonogenic survival of CTX-sensitive cells, which was more pronounced after stimulation with EGF compared to AREG. While the effect of EGF stimulation on clonogenic survival was significant for SCC-9 and UT-SCC-9P cells, only a trend was observed for FaDu. Conversely, CTX significantly blocked clonogenic survival in all CTX-sensitive cells. The inhibitory effect was more pronounced in spheroid compared to monolayer cultures. As seen also for apoptosis, there was no influence of EGF or CTX on the clonogenic potential of CTX-resistant UT-SCC-9R cells.

## Discussion

Using spheroid culture of HNSCC cell lines as a representative *in-vitro* model for CTC cluster formation and growth after loss of cell-matrix interactions contributing to the metastatic spread *in vivo*, [10] we here established an important role of EGFR activation for HNSCC cell survival and spheroid maintenance under such conditions. Spheroid formation leading to resistance to anoikis by autocrine EGFR stimulation could be mediated by the activation of different downstream components of the EGFR signalling pathway including upregulation of the hypoxia-inducible-factor-1 (HIF1) [29] or activation of the PI3K/AKT [30] as well as the MAPK pathway [18]. In support of a major role of MAPK, the activation of this pathway was shown to be required for spheroid formation [18]. Consistent with these previous studies, we observed that autocrine EGFR signalling through MAPK/ERK was maintained after loss of cell adherence in all HNSCC cells and that the strongest activation of MAPK/ERK was observed in the cell line with the highest capacity of spheroid formation. Moreover, exogenous stimulation of EGFR increased the number and volume of spheroids. Interestingly, differences in the effects of AREG and EGF on spheroid formation capacity were observed, indicating divergent molecular mechanisms influenced by these two EGFR ligands. In line with our observation, EGF was demonstrated to induce morphological changes in colon cancer cell lines whereas AREG had an effect only on cell proliferation [31].

The results from our *in-vitro* model are suggestive of EGFR being a master regulator of cell survival not only in the bulk tumour [32] but also in CTC-forming clusters. This conclusion is further supported by our previous observation that basal expression of EGFR can be detected in 100% and activated EGFR (pEGFR) in 55% of CTCs from HNSCC patients with locally advanced stage disease [25]. EGFR-expressing CTCs have also been detected in metastatic breast cancer patients as well as patients with non-small-cell lung cancers [33,34]. These data strongly suggest that EGFR might not only represent an attractive molecular target in the bulk tumour [35] but also in CTCs to prevent them from forming clusters. In favor of such a therapeutic approach is our previous observation that the radiation-induced increase in CTC numbers was less pronounced when radiotherapy was combined with CTX compared to chemotherapy [25]. However, addition of CTX to radiotherapy significantly improved local but not distant control in the Bonner trial [36]. This negative result could be explained by an enrichment of cells with primary or acquired resistance to CTX among CTCs. Generally, in primary tumours acquired resistance to anti-EGFR antibodies could result from compensatory mechanisms for reduced EGFR signaling [37] as well as genetic alterations in the EGFR-RAS-RAF-MEK signaling pathway or other receptor tyrosine kinases [38]. Several previous studies also revealed the hepatocyte growth factor receptor MET as a potential mediator of resistance to anti-EGFR targeted therapies [39–43]. MET activates intracellular signaling cascades including RAS-MAPK and PI3K-AKT pathways, NF-*κ*B and Wnt/GSK-3*β*/*β*-Catenin signaling [44]. Moreover, an increased expression of MET in combination with CD47 and EpCAM was shown to be correlated with a high tumorigenicity of CTC subpopulations [2,45]. Increased expression of MET has been described for CSCs and has a key role in cell growth, survival and metastasis [44]. Therefore, CTCs could contain a subpopulation of CSCs with intrinsically lower sensitivity to CTX treatment. CSC characteristics have indeed been described for CTCs, and it was postulated that it is their stemness feature which enables them to survive in peripheral blood as single-dissociated cells or small spheroids [46–48] (reviewed in ref. 4). Thus, targeting also key regulators in MET activated pathways might help improving distant control in HNSCC. These aspects are currently under investigation in our laboratory.

The poor distant control by CTX treatment in HNSCC patients might be alternatively explained by the occurrence of CTCs in peripheral blood of patients as tightly packed spheroids [12]. Thus, resistance to CTX could simply result from a reduced accessibility of the antibody to inner spheroid layers where the true metastasis-initiating cells might be located. Recently, the in-depth characterization of tumour cell spheroids from peripheral blood of patients with solid cancers revealed heterogeneous cell layers, with cells displaying a cancer stem cell (CSC)-like phenotype in the center and cells with a more differentiated phenotype at the spheroid surface [12]. Moreover, in CSCs high expression of efflux pump genes is known to cause multidrug resistance, especially for chemotherapeutic agents [49,50]. In agreement with this, another study demonstrated resistance to anoikis and cytotoxic drugs for CTM rather than for solitary CTCs in SCLC [17]. If confirmed in our *in-vitro* model, strategies to target the mechanisms involved in spheroid aggregation and maintenance might represent an attractive combinatory approach to improve the anti-metastatic efficacy of EGFR blockade as well as chemotherapy in CTC clusters.

In conclusion, HNSCC cells lacking cell-matrix interactions require mechanisms including EGFR signalling to form cell clusters to be protected from anoikis-like cell death. An anti-metastatic therapeutic approach directed against CTCs or CTC clusters should thus include EGFR as a molecular target. However, other mechanisms such as the factors responsible for cell aggregation in tightly packed spheroids and/or the acquisition of a CSC phenotype in such structures might also contribute to resistance from anoikis. Future studies focusing on a more detailed characterisation of the architecture and physiological characteristics of spheroids *in vitro* and *in vivo* are required in order to optimize current treatment concepts for a better distant tumour control in HNSCC.

## Supporting Information

S1 Fig Validation of resistance and sensitivity to cetuximab in different HNSCC cell lines. UT-SCC-9P was chronically treated with increasing cetuximab concentrations. The CTX-resistant phenotype of the subclone UT-SCC-9R was confirmed by assessment of the inhibitory effect of CTX treatment on clonogenic cell survival in UT-SCC-9R compared to the parental cell line UT-SCC-9P, FaDu and SCC-9. Bars show the mean survival fractions (SF) ± standard deviation for the different CTX concentrations and cell lines from three independent experiments.
(TIFF)

## Abbreviations

AREG: amphiregulin
CSC: cancer stem cells
CTCs: circulating tumour cells
CTM: circulating microemboli
CTX: cetuximab
EGF: epidermal growth factor
EGFR: epidermal growth factor receptor
FS: forced suspension
HNSCC: head and neck squamous cell carcinoma
M: monolayer
MTT: Methylthiazolyldiphenyl-tetrazolium bromide
NSCLC: non-small cell lung cancer
pEGFR: phospho-EGFR
PI: Propidium iodid
poly-HEMA: Poly(2-hydroxyethyl methacrylate)
SCLC: small cell lung cancer
SD: standard deviation
