# Supplementary figures and images for: Spheroid Culture of Head and Neck Cancer Cells Reveals an Important Role of EGFR Signalling in Anchorage Independent Survival
Source: PLoS One. 2016 Sep 19;11(9):e0163149. doi: 10.1371/journal.pone.0163149 (PMC5028019; doi:10.1371/journal.pone.0163149)

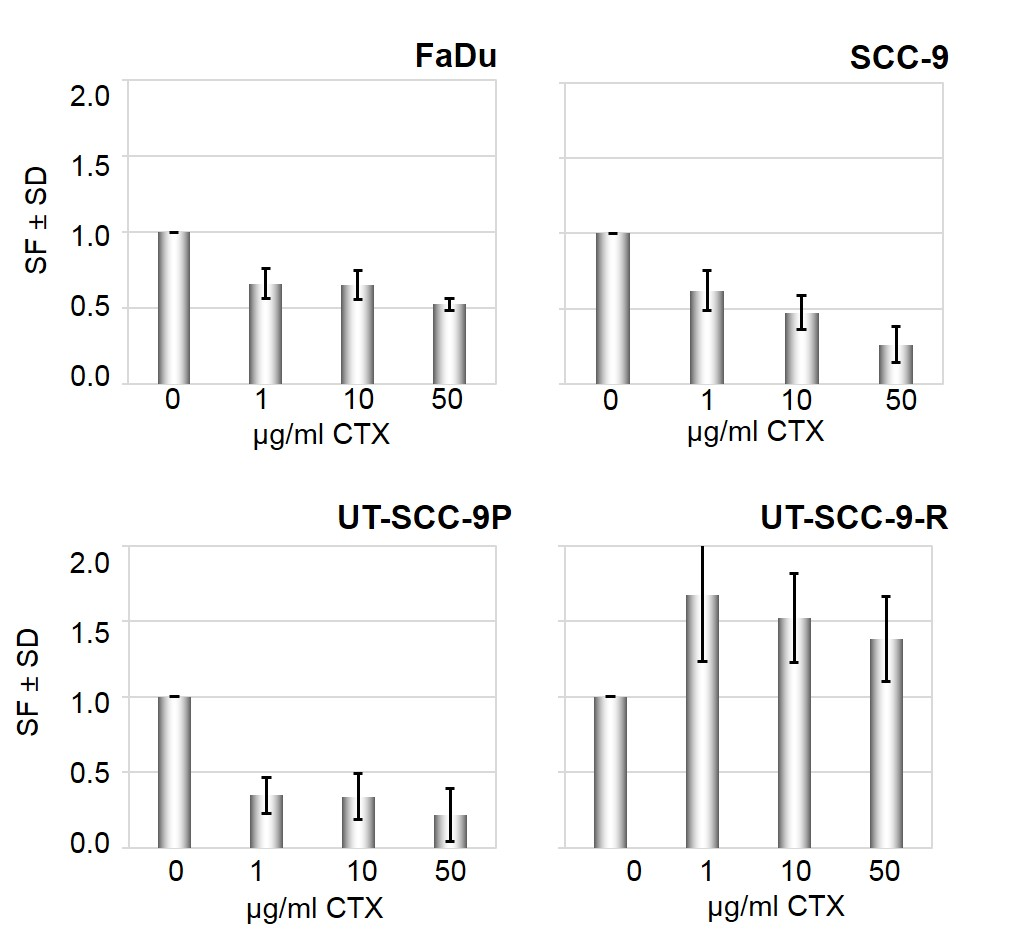

Supplement: S1 Fig — UT-SCC-9P was chronically treated with increasing cetuximab concentrations. The CTX-resistant phenotype of the subclone UT-SCC-9R was confirmed by assessment of the inhibitory effect of CTX treatment on clonogenic cell survival in UT-SCC-9R compared to the parental cell line UT-SCC-9P, FaDu and SCC-9. Bars show the mean survival fractions (SF) ± standard deviation for the different CTX concentrations and cell lines from three independent experiments. (TIFF) [file pone.0163149.s001.tiff]
